# Lupulin as an Anaphrodisiac

**Published:** 1855-04

**Authors:** 


*Lupulin as an Anaphrodisiac.*—Lupulin, or the active principle of hops, is possessed of powerful sedative effects on the generative functions. This was first entertained by Debout, but more recently Zambaco has published a paper on its anaphrodisiac virtues (*Bull. de Therap.* 30th August, 1854), in which he more than confirms this author's observations. Zambaco administered the medicine in doses varying from 1 to 16 grammes, and he never found sickness or constitutional disturbance attend its use. He has recorded the history of eight cases of painful erections, following gonorrhea, in which it was most successfully employed as a sedative. He affirms that in four-fifths of the cases it allays the morbid erethism, and prevents chordee. It answers this purpose much better than camphor, which often irritates the digestive functions, and fails to produce the desired effect. Besides being possessed of sedative and anti-blenorrhagic properties, which depend on its essential oil and resinous principle, lupulin contains a bitter element which acts as an admirable tonic. Zambaco has seen lupulin given, *as a tonic*, to strumous patients, with the best effects, the appetite becoming improved, and the digestive organs strengthened.—*Edin. Monthly Journal of Medicine.*

The administration of lupulin as an anaphrodisiac, was proposed by Dr. Wm. Byrd Page, of this city, in the year 1849. In his remarks upon it, he states that he had successfully used it as a remedy to prevent nocturnal erections, more than two years previous to the publication of his article. See Med. Examiner, Vol. v. May, page 284.—Editor.

